# PTHrP-associated hypercalcemia in gynecologic malignancies: a scoping review

**DOI:** 10.1007/s00404-026-08488-y

**Published:** 2026-06-17

**Authors:** Shara Maria Bohne, Florian Wegwitz, Judith Kruse-Wieczorek, Laura Langer, Julia Gallwas, Philipp Ströbel, Mir Fuad Hasanov

**Affiliations:** 1 Department of Gynecology and Obstetrics, University Medical Center Göttingen, Göttingen, Germany; 2 Department of Gynecology and Obstetrics, Laboratory for Molecular Gynecology, University Medical Center Göttingen, Göttingen, Germany; 3 Institute of Pathology, University Medical Center Göttingen, Göttingen, Germany

**Keywords:** Parathyroid hormone-related peptide (PTHrP), Humoral hypercalcemia of malignancy (HHM), Gynecologic malignancies, Paraneoplastic syndrome

## Abstract

**Purpose:**

Parathyroid hormone-related peptide (PTHrP) is a peptide hormone that shares structural similarity with parathyroid hormone (PTH) and binds to the same receptor, leading to increased calcium levels. Humoral hypercalcemia of malignancy (HHM) is typically mediated by tumor-derived PTHrP and accounts for the majority of cases of malignancy-associated hypercalcemia, whereas it is rarely observed in benign conditions. The purpose of this study was to systematically review the literature to map and provide a structured and transparent overview of reported cases of HHM in malignant gynecologic tumors.

**Methods:**

A systematic literature search was conducted in PubMed/MEDLINE and Web of Science, complemented by manual reference screening and additional searches using Google Scholar, identifying relevant articles published between 1973 and 2025. Clinical and biochemical characteristics were extracted and analyzed descriptively.

**Results:**

A total of 51 reported cases were identified, including 29 ovarian, 12 uterine, 5 vulvar, and 5 cervical tumors. Among the 29 cases in which serum PTHrP was measured, levels were elevated in 28 patients. In the remaining cases, suppressed PTH levels, exclusion of alternative causes, or indirect evidence from tissue immunohistochemistry (IHC) supported PTHrP involvement. Preoperative calcium levels ranged from 10.5 to 21.3 mg/dL. Following tumor resection, both PTHrP and calcium levels declined and normalized in most patients.

**Conclusion:**

These findings suggest that although rare, PTHrP-mediated hypercalcemia represents a clinically relevant paraneoplastic phenomenon in gynecologic malignancies. Prospective studies with standardized biochemical and tissue-based assessment are needed to clarify its epidemiology and clinical significance.

**Supplementary Information:**

The online version contains supplementary material available at 10.1007/s00404-026-08488-y.

## Introduction

Hypercalcemia is a recognized metabolic complication in patients with malignancy and is associated with significant morbidity and mortality [1, 2]. Parathyroid hormone-related peptide (PTHrP) plays a central role in the development of Hypercalcemia of Malignancy (HCM). While PTHrP levels are typically low or undetectable in healthy individuals [3] and benign tumors, PTHrP expression and secretion have been reported in several malignancies, most notably cancers of the lung, head and neck, esophagus, breast, and kidney [4, 5]. In gynecologic tumors (ovary, uterus, vulva, and cervix), PTHrP involvement has been reported [6] though it is considered rare in the literature [7], with evidence primarily limited to case reports. A review published in 2009 [6] provides a summary of the available cases at that time. However, since then, additional case reports have been published, and a comprehensive, structured evaluation of the evolving evidence is lacking. The available literature remains heterogeneous, with variable levels of biochemical confirmation and inconsistent reporting of clinical and laboratory findings. Given this heterogeneity and the predominance of case reports, a scoping review approach was considered appropriate to map and systematically summarize the existing evidence. The aim of this scoping review was therefore to provide a structured and transparent overview of reported cases of PTHrP-associated hypercalcemia in gynecologic malignancies. In addition to updating the available evidence, this study evaluates pre- and postoperative biochemical parameters and stratifies cases according to the level of diagnostic confirmation.

### Biological and molecular characteristics of PTHrP

PTHrP is encoded by the *PTHLH* gene that is located on chromosome 12 at position 12p11.22 and is part of the parathyroid hormone (PTH) gene family [8, 9]. Its transcripts encode precursors typically ranging from 139 to 173 amino acids in humans depending on the specific isoform and alternative splicing variants [3]. Its structure closely resembles that of PTH, which can be explained by the fact that both PTH and PTHrP are encoded by related genes [8, 9]. Eight of the first 13 amino acids are identical to those of the natural PTH.

The similarity in structure allows PTHrP and PTH to interact with the same type 1 PTH/PTHrP receptor (PTH1R) and thus to carry out a similar function [3, 4]. The length variation of the PTHrP products contributes to the generation of multiple bioactive fragments, some of which retain the ability to bind and activate the PTH/PTHrP receptor, thereby mediating diverse physiological and pathological effects [8].

PTHrP is a peptide hormone physiologically expressed in various tissues, including breast tissue, placenta, and vascular smooth muscle cells [3, 10]. Under physiological conditions, PTHrP fulfills several important functions, particularly in the context of reproduction and calcium homeostasis [3]. During pregnancy, this peptide is produced by the placenta, facilitating the transfer of sufficient calcium to the fetus, which is essential for proper bone growth [3, 11]. In the mammary glands, PTHrP plays a crucial role in maintaining calcium homeostasis during lactation, ensuring that the infant receives adequate calcium [3]. Additionally, PTHrP induces relaxation in smooth muscle cells, contributing to the dilation of blood vessels and thereby ensuring an adequate fetal blood supply during pregnancy [9, 10].

### PTHrP and malignancy

Its best-known pathological role is in humoral hypercalcemia of malignancy (HHM), a paraneoplastic syndrome characterized by elevated calcium levels due to tumor-associated PTHrP secretion [5, 12]. PTHrP acts as a mediator, increasing blood calcium levels by enhancing bone resorption and promoting calcium reabsorption in the kidneys [13]. It plays an important role in the growth and survival of the tumor by blocking pro-apoptotic signals, upregulating anti-apoptotic molecules, and promoting angiogenesis [8, 14]. HCM occurs in approximately 20–30% of patients with cancer [12, 15] although reported rates vary widely from 2 to 44% depending on tumor type, disease progression and study population [3–5, 16]. HCM can be categorized in four main groups based on the underlying mechanism [3–5, 12]: (I) Local osteolytic hypercalcemia (~ 20%), (II) HHM (~ 80%), (III) Hypercalcemia from 1,25-dihydroxyvitamin D-secreting lymphomas (< 1%), and (IV) Hypercalcemia from ectopic hyperparathyroidism (< 1%). In summary, while various pathways contribute to HCM, PTHrP-driven HHM is by far the most common [12].

## Methods

### Scoping review

A scoping review approach was chosen for this study, as it is particularly well suited to mapping the types and characteristics of available evidence on a given topic. This review was structured following methodological frameworks described by Arksey and O’Malley [17], Levac et al. [18], and recent guidance by the Joanna Briggs Institute (JBI) [19] and Mak & Thomas [20], and was reported in accordance with the PRISMA-ScR framework [21]. We applied the PCC framework (Population–Concept–Context) to guide the development of the research question and inclusion criteria [19]. This approach facilitates a structured overview of a heterogeneous evidence base, highlighting the range and nature of available reports while identifying areas where further research is warranted.

### Search strategy

A systematic search of the PubMed/MEDLINE database and Web of Science was conducted to identify relevant literature in accordance with the PRISMA-ScR guidelines [21]. The search was last updated in April 2026. A block-based search strategy was applied, combining terms related to PTHrP with terms describing gynecologic malignancies (Table 1). Additional terms related to calcium metabolism were used to refine the search. Synonyms within each search block were combined using the Boolean operator “OR”, and the different blocks were combined using “AND”. Searches were limited to articles published in English and German. In addition to database searching, supplementary searches were conducted through systematic screening of reference lists of included articles to account for potentially limited indexing and the dispersed nature of the literature. Furthermore, a search was performed using Google Scholar with the keyword combination “PTHrP gynecologic malignancy hypercalcemia”, and the first 100 results were screened for potentially eligible studies.

**Table 1:** Search Terms

Data Base	Search terms
PubMed/MEDLINE	("PTHrP" OR "parathyroid hormone related peptide" OR "PTHLH") AND ("sarcoma" OR "ovarian tumor" OR "ovarian cancer" OR "vulvar tumor" OR "vulvar cancer" OR "uterine tumor" OR "leiomyosarcoma" OR "endometrial stromal-sarcoma" OR "clear cell carcinoma" OR "small cell carcinoma" OR "cervical tumor" OR "cervical cancer") AND ("Hypercalcemia" OR "Humoral Hypercalcemia of malignancy" OR "calcium")
Web of Science	("PTHrP" OR "parathyroid hormone related peptide" OR "PTHLH") AND ("sarcoma" OR "ovarian tumor" OR "ovarian cancer" OR "vulvar tumor" OR "vulvar cancer" OR "uterine tumor" OR "leiomyosarcoma" OR "endometrial stromal-sarcoma" OR "clear cell carcinoma" OR "small cell carcinoma" OR "cervical tumor" OR "cervical cancer") AND ("Hypercalcemia" OR "Humoral Hypercalcemia of malignancy" OR "calcium")

### Eligibility criteria

We defined the following inclusion criteria: (1) Published case reports or clinical studies describing malignant gynecologic tumors (ovarian, vulvar, cervical, uterine) associated with HHM, and (2) Evidence of actual or probable PTHrP involvement. Exclusion criteria involve cases of hypercalcemia attributable to local osteolytic processes (e.g., bone metastases), primary hyperparathyroidism, vitamin D-mediated causes, or medication-related hypercalcemia.

HHM was considered present if at least one of the following criteria was met: (I) Elevated PTHrP levels in serum; (II) Demonstration of PTHrP expression in tumor tissue e.g. by immunohistochemistry (IHC); (III) Notable laboratory findings suggestive of PTHrP-mediated hypercalcemia (e.g., suppressed PTH levels).

### Study selection

**Fig. 1:**
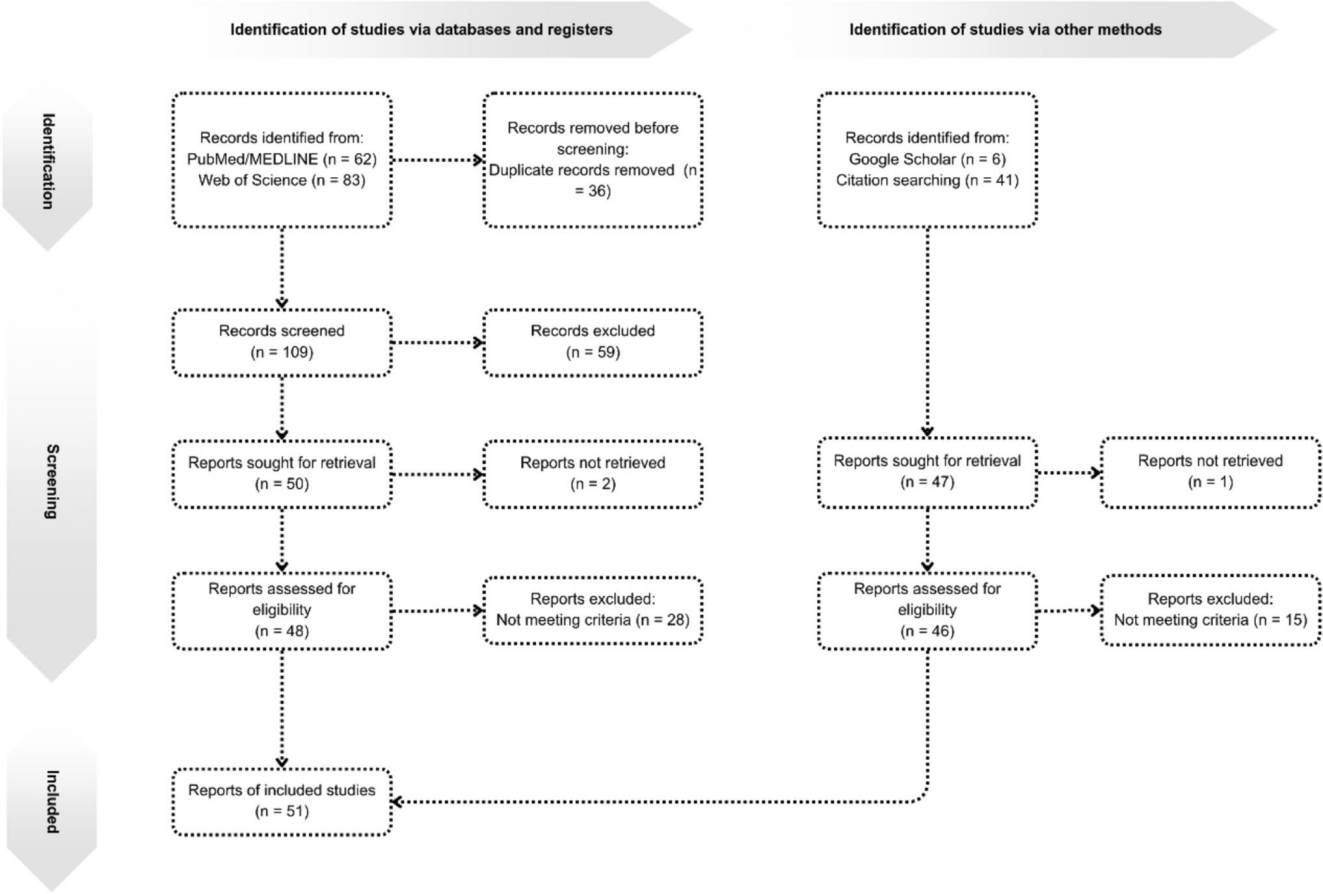
PRISMA flow diagram of study selection

Study selection was conducted in two steps: (I) Title/ abstract screening based on the eligibility criteria; (II) Full-text review to confirm inclusion. Screening was performed independently by two reviewers. In case of disagreement, discrepancies were resolved through discussion until consensus was reached. The study selection process is illustrated in Fig. 1.

### Data extraction

A comprehensive tabular overview of all included cases is provided in Online Resource 1. Data extracted included patient age, FIGO stage at presentation, histologic subtype, serum PTH and PTHrP levels (pre- and postoperatively), serum calcium levels (pre- and postoperatively), primary tumor treatment, recurrence-free survival, and overall survival. Survival time was reported in days for durations < 1 week, in weeks for durations < 1 month, and in months for longer follow-up periods. When multiple measurements of calcium, PTH, or PTHrP levels were reported, the highest preoperative value and the first postoperative value were recorded. To ensure a clear and transparent presentation of the data, cases were stratified into three groups based on the level of evidence for PTHrP involvement: (1) Serum-confirmed PTHrP elevation, (2) Exclusively tissue-confirmed PTHrP expression by IHC, and (3) Presumed PTHrP-mediated hypercalcemia. Within each group, cases were further categorized according to the anatomical origin of the malignancy (ovarian, uterine, cervical, or vulvar). HHM at recurrence was denoted with a superscript “R”. Although FIGO stage IIC was removed in the 2014 revision, we retained the originally reported staging for consistency with historical publications. Not all publications reported complete biochemical data; in some cases, studies were included even if PTHrP was not directly measured but was considered the most likely cause of hypercalcemia based on the clinical context.

### Assessment and interpretation of PTHrP levels

Due to substantial heterogeneity in PTHrP assay methodologies and reported reference ranges across studies, no uniform cut-off value was applied.

When reference ranges were reported, these were typically based on immunoassay-derived upper limits (e.g., approximately < 2.0 pmol/L [22]), whereas more recent mass spectrometry-based studies reported higher assay-specific reference ranges. Reference ranges reported in the results are provided for descriptive orientation only. PTHrP concentrations were classified as elevated or normal according to the definitions used in the original publications.

### Unit conversion

For comparability across case reports, PTH values were standardized to pg/mL and PTHrP values to pmol/L. Most case reports already reported PTH in pg/mL and PTHrP in pmol/L, requiring no conversion. In cases where conversion was necessary, PTH concentrations reported in pmol/L were converted to pg/mL using a conversion factor based on the molecular weight of intact PTH (1–84) of 9430 Daltons (1 pmol/L = 9.43 pg/mL). PTHrP concentrations reported in pg/mL were converted to pmol/L using a conversion factor based on the molecular weight of PTHrP (1–84) of approximately 9425 Daltons (1 pg/mL = 0.106 pmol/L).

## Results

A total of 51 cases were identified and included in the analysis, published between 1973 and 2025. We uncovered 17 additional cases since the last review in 2009 [6]. Among the 51 malignant tumors, there are 29 ovarian, 12 uterine, 5 vulvar and 5 cervical neoplasms. Clear cell carcinoma and squamous cell carcinoma were identified as the predominant histological subtypes. Serum PTHrP was assessed in 29 cases and was elevated in 28. In three cases, PTHrP expression was additionally confirmed by IHC in tumor tissue alongside positive serum measurements [23–25]. In one additional case, PTHrP expression was detected exclusively by IHC in a metastatic lymph node, without available serum measurements [26]. In 21 cases, neither serum PTHrP nor IHC was performed. Among these, 18 patients had normal or suppressed PTH levels. Across all groups, PTH was measured in 44 cases and was normal or suppressed in 43. Postoperative calcium levels were measured in 27 cases, with PTHrP levels assessed in 15 of these. Following tumor resection, both serum calcium and PTHrP levels decreased markedly. In the majority of patients, both parameters returned to the normal range. In addition, recurrences or rapid tumor progression triggered a subsequent rise in both calcium and PTHrP levels [26–29].

### Descriptive analysis

**Fig. 2:**
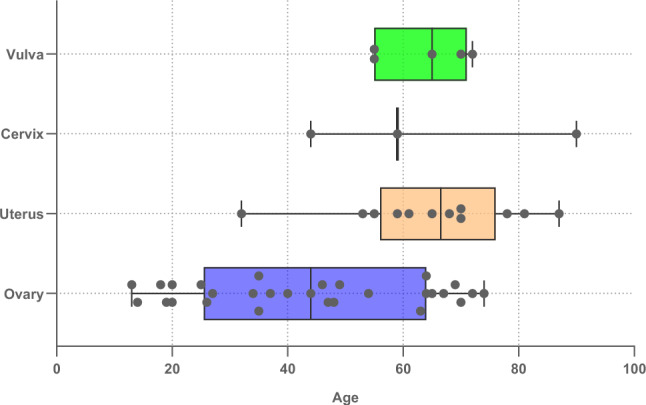
Mean age by gynecologic tumor type

This analysis demonstrates a difference in age distribution across tumor types. Ovarian tumors occurred at substantially younger ages (median 44 years; *n* = 29) compared with uterine, vulvar and cervical tumors, which presented predominantly in older patients (uterus: median 66.5 years, *n* = 12; vulva: median 65 years, *n* = 5; cervix: median 59 years, *n* = 3/5). Overall, this corresponds to a clinically relevant ~ 20-year difference in median age between ovarian and uterine/vulvar tumor entities (Fig. 2).

Ovarian tumors demonstrate the broadest age spectrum of all tumor types. The range extends from pediatric cases (e.g., a 13-year-old patient) [30] to older adults (e.g., a 74-year-old patient) [31].

**Fig. 3:**
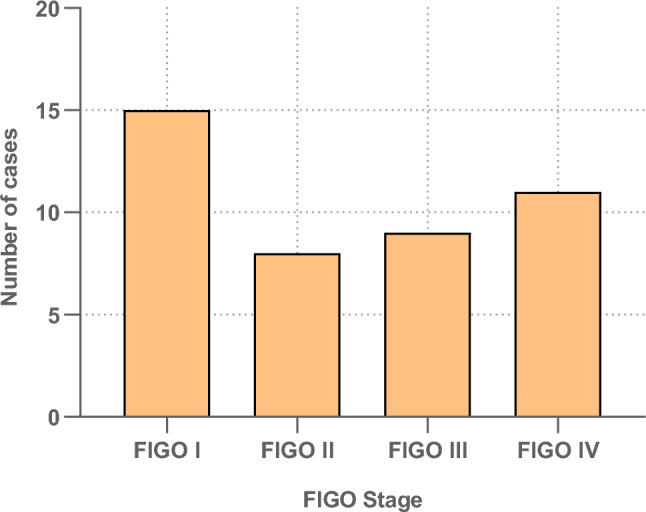
Distribution of reported cases of PTHrP-associated hypercalcemia by FIGO stage

Across the entire cohort of recorded tumors, FIGO Stage I was the most frequent clinical stage, representing about one-third of all valid cases (Fig. 3). Advanced stages (III and IV combined) accounted for nearly half of the patients, indicating a balanced distribution between early and late diseases. A small proportion of cases (15.7%) had missing stage information.

### Ovarian malignancies

The ovarian malignancies constitute the largest group, with 29 documented case reports. We found a range of tumor types, among which the clear cell carcinoma and the dysgerminoma were the most commonly associated with HHM (Table 2). Median calcium levels were 14 mg/dL (range: 10.5–20.9 mg/dl, reference range: 8.9–10.1 mg/dl). Serum PTHrP was measured in 16 cases and was elevated in all of them. Median serum PTHrP levels were 19.5 pmol/L (range: 2.3–29.200 pmol/L, reference range: < 2 pmol/L). In one additional case, PTHrP was only detected by IHC in a metastatic lymph node [26]. In 23, PTH levels were reported as physiological or decreased. In most cases, PTH values ranged from < 1 to 14 pg/mL (reference range: 15–65 pg/mL). One case with a PTH level of 143 pg/mL was also classified as normal as the reference range reported in that study was 150–500 pg/mL.

**Table 2:** Distribution of ovarian tumors according to WHO classification

WHO category	Tumor entity	Number
Epithelial tumors	Clear cell carcinoma	13
	Serous carcinoma	4
	Small cell carcinoma	3
	Mucinous carcinoma	1
	Squamous cell carcinoma	1
	Metastatic adenocarcinoma	1
Germ cell tumors	Dysgerminoma	5
Sex cord stromal tumors	Juvenile granulosa cell tumor	1
Total		29

### Uterine malignancies

The uterine malignant neoplasms represent the second largest group, comprising 12 tumors, with clear cell carcinoma being the most common subtype (Table 3).

**Table 3:** Distribution of uterine tumors according to WHO classification

WHO category	Tumor entity	Number
Endometrial cancers	Clear cell carcinoma	5
	Endometrioid adenocarcinoma	2
	Dedifferentiated endometrial carcinoma	1
	Endometrial serous papillary carcinoma	1
	Carcinosarcoma	1
Mesenchymal tumors	Metastatic epithelioid leiomyosarcoma	1
	High-grade endometrial stromal sarcoma	1
Total		12

The median calcium level was 14.02 mg/dL (range: 11.4–21.3 mg/dL, reference range: 8.9–10.1 mg/dL). PTHrP was measured in 10 cases and was elevated in the majority of patients. In one case, PTHrP levels were within the assay-specific reference range but declined following tumor resection in parallel with normalization of serum calcium [32].

Median PTHrP serum levels were 5.55 pmol/L (range: 1.4–4550 pmol/L, reference range: < 2 pmol/L). PTH levels were reported as normal or below the respective reference range in all cases, except for one case of endometrioid carcinoma in which PTH was elevated concomitantly with PTHrP [29]. In the remaining cases, PTH values ranged from < 2.5 to < 7 pg/mL (reference range: 15–65 pg/mL). Two additional cases showed PTH values of 67 pg/mL and 88 pg/mL; however, both were considered within the normal range according to the assay-specific reference intervals reported in the original studies (20–90 pg/mL).

### Cervical malignancies

Four of five patients with cervical malignancies had squamous cell carcinoma, and one had high-grade clear cell carcinoma. Serum PTHrP was measured in two cases and was elevated in both (one reported value: 9.3 pmol/L, reference range: < 2.0 pmol/L). PTH levels were described as normal or suppressed in all cases; numerical values were available in two patients (12 and 11.9 pg/mL, reference range: 15–65 pg/mL). Median serum calcium levels were 15.8 mg/dL (range: 13.9–18 mg/dL, reference range: 8.9–10.1 mg/dL).

### Vulvar malignancies

Among the five patients with vulvar neoplasms, squamous cell carcinoma was the predominant histologic subtype. PTHrP serum levels were assessed in one patient and were elevated in that case at 23.2 pmol/L (reference range: < 2 pmol/L). In all three cases in which PTH was measured, concentrations were decreased or within the normal range. One value was measured at 6.2 pg/mL (reference range: 15–65 pg/ml), while the remaining cases were reported as normal or undetectable in the original studies. The median serum calcium level was 16.35 mg/dL (range 13.1–17.0 mg/dL, reference range: 8.9–10.1 mg/dL).

### Serum calcium levels before and after surgery

After surgical intervention, serum calcium levels demonstrated a marked and consistent decline, with the majority of cases normalizing to values near or below 10 mg/dL (reference range: 8.9–10.1 mg/dL).

**Fig. 4:**
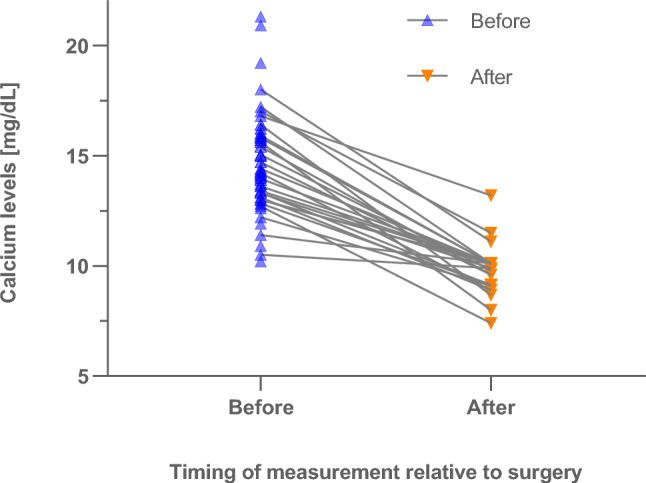
Paired dot plot of serum calcium before and after surgery

In contrast, preoperative peak serum calcium levels frequently exceeded the upper normal limit, with several patients exhibiting substantial hypercalcemia (often 14–20 mg/dL) (Fig. 4).

### Serum PTHrP levels before and after surgery

**Fig. 5:**
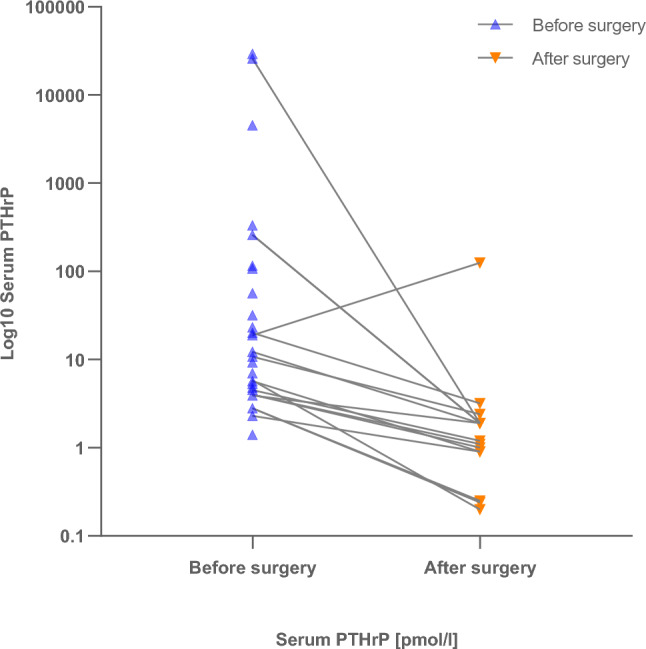
Paired dot plot of log10 serum PTHrP before and after surgery

Surgical treatment was associated with a consistent decrease in serum PTHrP levels. Preoperative PTHrP concentrations showed considerable interindividual variability with a long right-skewed distribution, including several markedly elevated values over 4000 pmol/L (reference range: < 2 pmol/L) [25, 28, 33]. After surgery, most patients demonstrated normalized or near-undetectable serum PTHrP levels (Fig. 5). One apparent outlier showed an increase in PTHrP. This value was reported as a postoperative measurement in the original publication but was obtained at a later time point rather than immediately after surgery [34]. In this patient, serum calcium initially decreased postoperatively (from 14.7 to 9.6 mg/dL) but increased again to 13.7 mg/dL within 7 days, at which time PTHrP was measured and found to be elevated.

## Discussion

This scoping review identified a consistent association between cases of humoral hypercalcemia in gynecologic malignancies and elevated PTHrP levels, particularly in ovarian and uterine tumors. The most frequently reported histological subtypes were clear cell carcinoma and squamous cell carcinoma. These findings are consistent with the review published in 2009 [6] and with previous reports showing that PTHrP-associated hypercalcemia is most commonly seen in these histological entities [35–37]. The broad age spectrum observed in ovarian tumors may be explained by the marked heterogeneity of ovarian neoplasms. Epithelial, germ cell, and cord stromal tumors differ substantially in their age distribution [38], which may account for the wide range of PTHrP-associated cases.

In contrast to the review published in 2009 [6], which primarily reported single time-point measurements, our analysis evaluated pre- and postoperative levels of PTH, PTHrP and calcium, allowing for the assessment of temporal changes following tumor resection. The observed pattern is consistent with the underlying pathophysiology: PTHrP binds to the same PTH1 receptor as endogenous PTH, leading to increased serum calcium levels and subsequent suppression of PTH secretion via physiological negative feedback [13]. The observed parallel decline in PTHrP and calcium levels following tumor removal, accompanied by recovery of suppressed PTH, supports a functional role of tumor-derived PTHrP in the pathogenesis of hypercalcemia in these cases.

One apparent outlier in the graphical analysis showed an increase in PTHrP levels following surgery in a 20-year-old patient with mucinous ovarian carcinoma (FIGO stage IIA) [34]. This finding does not represent a true immediate postoperative increase, as indicated in the original case report, but can be explained by the timing of measurement and the clinical course. In this case, PTHrP was not assessed when serum calcium levels had initially decreased directly after surgery, but only after a subsequent rise in calcium levels 7 days later, thus representing a follow-up measurement. The surgical approach was limited to a right salpingo-oophorectomy, likely resulting in residual tumor burden. The subsequent course was marked by rapid disease progression, including the development of pseudomyxoma peritonei (PMP) and invasion of adjacent structures, ultimately leading to the patient’s death within 30 days of hospitalization. The secondary increase in PTHrP levels is therefore most consistent with ongoing or progressive tumor activity. Similar patterns were observed in a subset of cases with serial measurements [26–29], in which PTHrP levels rose with tumor progression and declined after resection. These observations suggest that PTHrP secretion may not only contribute to hypercalcemia but also reflect changes in tumor activity over time.

Multiple preclinical and clinical studies support the role for PTHrP in driving tumor progression and metastasis. Experimental mouse models have demonstrated that PTHrP accelerates tumor growth, shortens tumor latency, and promotes metastatic spread, whereas genetic deletion or antibody-mediated inhibition of PTHrP results in reduced tumor growth and fewer metastases [39]. PTHrP has been implicated in the regulation of tumor cell dormancy through autocrine and intracrine mechanisms. Increased PTHrP expression is associated with reduced expression of dormancy-related genes and a more proliferative tumor cell phenotype, consistent with the development of clinically overt metastases [40, 41]. More broadly, PTHrP expression has been associated with increased proliferation, reduced apoptosis, angiogenesis, and induction of epithelial–mesenchymal transition (EMT), particularly in aggressive tumor subtypes such as triple-negative breast cancer [14, 40]. Overall, PTHrP exerts domain-dependent and context-specific effects in cancer. While numerous experimental studies support a role for PTHrP in promoting tumor growth and metastasis, particularly in the bone microenvironment, some clinical and preclinical data suggest that PTHrP expression in primary tumors may be associated with less aggressive disease [42].

While these findings highlight the diverse and context-dependent effects of PTHrP in tumor biology, the molecular mechanisms regulating its expression remain incompletely understood. In this context, recent evidence suggests that hypoxia-inducible factor 2 (HIF2) may act as an upstream regulator of *PTHLH* transcription.

In renal cell carcinoma models, HIF2-driven activation of *PTHLH* has been shown to increase tumor-derived PTHrP secretion and contribute to systemic manifestations, such as cachexia and hypercalcemia. These findings indicate that hypoxia-associated signaling pathways may represent an additional mechanism regulating PTHrP production in malignant tumors and highlight the HIF2–PTHrP axis as a potentially targetable pathway [43, 44].

### Limitations

This study has several limitations that should be considered when interpreting the findings. (I) The overall number of reported cases remains small, and the available evidence is largely derived from individual case reports, limiting the generalizability of the results. In addition, the literature is subject to reporting bias, with a likely overrepresentation of severe or clinically striking cases. Several factors may have contributed to this low number: PTHrP was not biochemically characterized until the late 1980s; its relevance remains underrecognized in clinical practice and it is therefore rarely included in diagnostic workups for malignancy-associated hypercalcemia. (II) There is heterogeneity in the biochemical assessment of PTHrP. Assay methodologies, reference ranges, and reporting standards vary widely across studies, particularly between older immunoassay-based measurements which typically reported lower upper reference limits around 2.0 pmol/L [22] and more recent LC–MS/MS approaches with sex-specific reference ranges of approximately 0.6–3.3 pmol/L in women and 0.5–2.2 pmol/L in men [45]. These differences preclude the use of a uniform cut-off value and complicate direct comparison between studies. (III) The completeness of biochemical data is limited. In 22 cases, PTHrP was not directly measured, and its involvement was inferred from indirect findings and exclusion of alternative causes of hypercalcemia.

Beyond these limitations, important gaps remain regarding the biological role of PTHrP in gynecologic malignancies. To date, only a few, predominantly older studies have directly investigated PTHrP expression in tumor tissue. These studies reported positive immunohistochemical staining in invasive squamous cell carcinomas of the cervix and in ovarian small cell carcinoma [46, 47]. However, comprehensive and contemporary studies systematically assessing the prevalence and clinical significance of PTHrP expression in gynecologic tumors are still lacking. It therefore remains unclear how frequently PTHrP is expressed in these tumors overall and to what extent this expression is associated with clinically manifest hypercalcemia. Notably, PTHrP expression and hypercalcemia do not necessarily occur simultaneously as PTHrP has been detected in primary tumors of normocalcemic patients, with hypercalcemia developing only later during metastatic disease [24]. These observations further emphasize the need for systematic and standardized investigations to better define the role of PTHrP in gynecologic malignancies.

## Conclusion

PTHrP-associated hypercalcemia in gynecologic malignancies is a rare but recurrently reported phenomenon, particularly in ovarian and uterine tumors. Our findings confirm a consistent association between PTHrP and humoral hypercalcemia and demonstrate a characteristic postoperative decline in both PTHrP and calcium levels, supporting a tumor-driven mechanism. However, the true prevalence and clinical significance of PTHrP expression remain unclear, likely due to underrecognition and heterogeneous diagnostic approaches. Future prospective studies with standardized biochemical and tissue-based assessments are needed to better define its epidemiology, biological role, and potential clinical relevance.

## Supplementary Information

Below is the link to the electronic supplementary material.

Supplementary file1 (DOCX 50 KB)

## Data Availability

No new datasets were generated. All data analyzed in this study are derived from previously published studies cited in the reference list and are summarized in Online Resource 1.
