# Supplementary material for: PTHrP-associated hypercalcemia in gynecologic malignancies: a scoping review
Source: Arch Gynecol Obstet. 2026 Jun 17;313(1):205. doi: 10.1007/s00404-026-08488-y (PMC13275765; doi:10.1007/s00404-026-08488-y)
Supplement: Supplementary file 1 — Supplementary file1 (DOCX 50 KB) [file 404_2026_8488_MOESM1_ESM.docx]

**Online Resource 1.** Reported cases of Humoral Hypercalcemia of Malignancy in gynecologic malignancies stratified by level of diagnostic confirmation

| Reference | Year of pub-lication | Age [years] | Initial stage | Reported histologic type | Serum PTH [pg/mL] before surgery  [15-65] | Serum PTH [pg/mL] after surgery  [15-65] | Serum PTHrP [pmol/L] before surgery  [<2] | Serum PTHrP [pmol/L] after surgery  [<2] | Peak Serum Ca2+ before surgery [mg/dl]  [8,9-10,1] | Serum Ca2+ after surgery [mg/dl]  [8,9-10,1] | Initial treatment | Outcome  (time) |
| --- | --- | --- | --- | --- | --- | --- | --- | --- | --- | --- | --- | --- |
| Group 1: Serum-confirmed PTHrP elevation | | | | | | | | | | | | |
| *Ovarian tumors* | | | | | | | | | | | | |
| Agarwal et al. | 2013 | 65 | IV | Papillary serous cystadenocar-cinoma | NR | NR | **116** | NR | **16^R^** | NR | Sx | RFS 120 months, OS >120 months |
| Agarwal et al. | 2013 | 47 | IIIC | Metastatic adenocarcinoma | NR | NR | **108** | NR | **15 ^R^** | NR | NR | NR |
| Benit et al. | 2006 | 74 | IA | Clear cell adenocarcinoma | **3** | **28** | **4.5** | **<1.3** | **12.83** | **9.14** | Sx, CTH | RFS and OS >12 months |
| Futagami et al. | 2010 | 64 | IA | Clear cell adenocarcinoma | **14** | **135** | **25900** | **normalized** | **18** | **11.1** | Sx | RFS and OS >2 weeks |
| Hoekman et al. | 1991 | 70 | IA | Clear cell adenocarcinoma | **<9.43** | **20.8** | **20.1** | **3.18** | **13.32** | **9.11** | Sx | NR |
| Hwang et al. | 2006 | 35 | IC | Clear cell adenocarcinoma | **12.8** | NR | **12.2** | **normalized** | **13.6** | **normalized** | Sx, CTH | RFS and OS >6 months |
| Inoue et al. | 1995 | 18 | IIIC | Dysgerminoma | NR | **normalized** | **258.9** | **normalized** | **14.4** | **normalized** | Sx, CTH | RFS and OS >30 months |
| Kitazawa et al. | 1997 | 67 | IV | Clear cell adenocarcinoma | **11** | NR | **332, additionally detected by IHC** | NR | **15.7 ^R^** | NR | Sx, CTH | RFS and OS 8 months |
| Koshiyama et al. | 1999 | 48 | IIC | Clear cell adenocarcinoma | NR | NR | **29200** | NR | **20.9 ^R^** | NR | Sx | RFS 18 months, OS 19 months |
| Ma et al. | 2018 | 63 | IIIC | Clear cell adenocarcinoma | **8.96** | NR | **12.2** | NR | **15.4** | **9.6** | Sx, CTH | RFS 5 months, OS 7 months |
| Montebello et al. | 2021 | 37 | NR | Small cell carcinoma (hypercalcaemic type, SCCOHT) | **<5** | **30** | **5.7** | **<1** | **15.9** | **<10.2** | Sx, CTH, XRT | RFS and OS >3 weeks |
| Schweiger and Hsiang | 2002 | 13 | IA | Small cell carcinoma | **<10** | NR | **4.82** | NR | **13.4** | **normalized** | SX, CTH | RFS 13 months, OS 36 months |
| Suwaki et al. | 2006 | 46 | IIC | Clear cell adenocarcinoma | **< 5** | **normalized** | **10.8** | **<2.5** | **13.3** | **<10** | Sx, CTH | RFS 36 months, OS >60 months |
| Takeuchi et al. | 2000 | 72 | III | Squamous cell carcinoma | **6** | NR | **2.3** | **<1** | **10.5** | **<10** | Sx, CTH, XRT | RFS 8 months, OS 17 months |
| Tsunematsu et al. | 2000 | 49 | IIIC | Clear cell adenocarcinoma | **2.83** | NR | **259** | **normalized** | **13** | **normalized** | Sx, CTH | RFS 3 months |
| Yeom et al. | 2020 | 20 | IIA | Mucinous carcinoma | **4.3** | NR | **18.9** | **125** | **14.7** | **9.6** | Sx | OS 1 month |
| *Uterine tumors* | | | | | | | | | | | | |
| Hutchesson et al. | 1993 | 59 | IB | Clear cell carcinoma | **<4.7** | NR | **2.8** | **<0.25** | **13.95** | **normalized** | Sx | RFS and OS >9 months |
| Kinugasa et al. | 2006 | 32 | IB | Endometrioid adenocarcinoma | **6** | NR | **4550, additionally detected by IHC** | NR | **21.3 ^R^** | NR | Sx, CTH | RFS 5 months, OS 7 months |
| Motilal Nehru et al. | 2017 | 53 | IVB | High-grade endometrial stromal sarcoma | **3** | NR | **31.9** | NR | **19.2** | NR | Sx, CTH, XRT | OS < 12 months |
| Ogino et al. | 2025 | 55 | NR | Dedifferentiated endometrial carcinoma | **312** | **55** | **3.9** | **not detectable** | **17.2** | **normalized** | Sx | RFS 1 month, OS 3 months |
| Richey and Welch | 2008 | 65 | NR | Clear cell carcinoma | **<7** | NR | **5.3** | NR | **14.3 ^R^** | NR | Sx, CTH | OS 49,5 months |
| Sachmechi et al. | 1995 | 68 | IB | Endometrial serous papillary carcinoma | **<4.72** | **1254.19** | **5.8** | **<0,2** | **12.7** | **7.4** | Sx, CTH | RFS 24 months, OS >24 months |
| Tang et al. | 2003 | 61 | IV | Metastatic epitheloid leiomyosarcoma | **3.13** | NR | **1.4** | NR | **11.4 ^P^** | **undetactable** | Sx | RFS 120 months |
| Takamatsu et al. | 2014 | 70 | IB | Carcinosarcoma | **<5** | **57** | **4** | **<1.1** | **14.2** | **8.9** | Sx, CTH | RFS 11 months, OS >13 months |
| Visnyei et al. | 2012 | 81 | NR | Endometrioid carcinoma | **<2.5** | NR | **7** | NR | **11.9** | NR | no treatment | NR |
| Georges et al. | 2023 | 70 | NR | Clear cell carcinoma | **6.6** | NR | **56.7** | NR | **13.7** | NR | Sx, CTH, XRT | NR |
| *Cervical tumors* | | | | | | | | | | | | |
| Chau and Ahmed | 2024 | 44 | NR | High-grade clear cell carcinoma | **normal** | NR | **elevated** | NR | **15.8** | NR | CTH, XRT | OS > 1 week |
| Matsuoka et al. | 2024 | 90 | III | Squamous cell carcinoma | **12** | NR | **9.3** | NR | **14** | NR | no treatment | OS 3 months |
| *Vulvar tumors* | | | | | | | | | | | | |
| Bilenchi et al. | 2005 | 70 | NR | Squamous cell carcinoma | NR | NR | **23.2, additionally detected by IHC** | NR | **elevated** | NR | Sx | OS 3 days |
| Group 2: Exclusively tissue -confirmed expression (IHC-positive) | | | | | | | | | | | | |
| *Ovarian tumors* | | | | | | | | | | | | |
| Fujino et al. | 1992 | 34 | IV | Clear cell adenocarcinoma | **143 (ref. 150-500)** | NR | **detected by IHC in metastatic lymph node** | NR | **16.2 ^R^** | NR | Sx, CTH, XRT | OS 13 months |
| Group 3: Presumed PTHrP-mediated hypercalcemia | | | | | | | | | | | | |
| *Ovarian tumors* | | | | | | | | | | | | |
| Allan et al. | 1984 | 40 | IV | Papillary cystadenocarcinoma | **low** | NR | NR | NR | **15.8 ^P^** | **normalized** | Sx, CTH | OS >4 months |
| Allan et al. | 1984 | 54 | III | Papillary cystadenocar-cinoma | NR | NR | NR | NR | **10.9** | NR | Sx, CTH | OS 14 months |
| Allan et al. | 1984 | 35 | IA | Clear cell adenocarcinoma | **low** | NR | NR | NR | **12.6 ^R^** | NR | Sx | RFS 48 months |
| Allan et al. | 1984 | 64 | IIB | Clear cell adenocarcinoma | **low** | NR | NR | NR | **12.2 ^R^** | NR | Sx | RFS 24 months |
| Allan et al. | 1984 | 69 | IIA | Serous cystadenocar-cinoma | **low** | NR | NR | NR | **14 ^R^** | NR | Sx, XRT | RFS 48 months |
| Fleisch-hacker and Young | 1994 | 19 | IA | Dysgerminoma | **2** | **normalized** | NR | NR | **12.2** | **9.1** | Sx, CTH | RFS and OS >24 months |
| Lewin et al. | 2012 | 44 | IC | Clear cell adenocarcinoma | **<1** | NR | NR | NR | **13** | **10.1** | Sx, CTH | RFS and OS >2 months |
| Louvet et al. | 1989 | 20 | IIA | Seminoma | **low** | NR | NR | NR | **12.2** | NR | Sx, CTH | RFS and OS >12 months |
| Okoye et al. | 2001 | 14 | IA | Dysgerminoma | **5** | NR | NR | NR | **15.04** | **9.79** | Sx | OS >10 months |
| Piura et al. | 2008 | 25 | IC | Juvenile granulosa cell tumor | **<3** | NR | NR | NR | **15.6** | **8** | Sx, CTH | RFS and OS >10 months |
| Radhakrishnan et al. | 2001 | 26 | IIIC | Dysgerminoma | **low** | NR | NR | NR | **16.4** | **8.67** | Sx, CTH | RFS and OS >33 months |
| Wynn et al. | 2004 | 27 | I | Small cell carcinoma | NR | **6** | **NR** | **0.12** | **13.6** | **normalized** | Sx, CTH | RFS 12 months, OS >16 months |
| *Uterine tumors* | | | | | | | | | | | | |
| Hiller et al. | 1989 | 78 | IV | Clear cell carcinoma | **67 (ref. 20-90)** | NR | NR | NR | **14.1^R^** | NR | Sx | RFS 12 months, OS 15 months |
| Hiller et al. | 1989 | 87 | IIIC | Clear cell carcinoma | **88 (ref. 20-90)** | NR | NR | NR | **12.8^R^** | NR | Sx | RFS 48 months, OS >57 months |
| *Cervical tumors* | | | | | | | | | | | | |
| Gupta et al. | 2020 | 59 | NR | Squamous cell carcinoma | **11.9** | NR | NR | NR | **13.9** | NR | XRT | NR |
| Lacey and Morrow | 1979 | NR | IVB | Squamous cell carcinoma | **normal** | NR | NR | NR | **18** | NR | XRT | OS 2 weeks |
| Lacey and Morrow | 1979 | NR | IVB | Squamous cell carcinoma | **normal** | NR | NR | NR | **17.2** | NR | XRT | OS 1 month |
| *Vulvar tumors* | | | | | | | | | | | | |
| Nichols et al. | 1973 | 55 | II | Squamous cell carcinoma | NR | NR | NR | NR | **16.8** | **13.2** | Sx | RFS 2 weeks, OS >24 months |
| Niebyl et al. | 1975 | 72 | II | Squamous cell carcinoma | **undetactable** | NR | NR | NR | **17** | **11.5** | Sx, XRT | RFS and OS >12 months |
| Niebyl et al. | 1975 | 55 | IV | Squamous cell carcinoma | **normal** | NR | NR | NR | **13.1** | **<9** | Sx, XRT | RFS 6 months, OS 54 months |
| Sükür et al. | 2009 | 65 | IVa | Squamous cell carcinoma | **6.2** | NR | NR | NR | **15.9 ^R^** | NR | Sx, CTH, XRT | RFS 4 months, OS 5 months |
| Abbreviations: Sx= Surgery, CTH = Chemotherapy, XRT= Radiotherapy, NR= Not reported, OS= Overall survival, RFS= Recurrence free survival, IHC=Immunohistochemistry,  Superscript R= measured at recurrence, Superscript P= measured in primary lesion | | | | | | | | | | | | |
